# Bayesian Network in Structural Health Monitoring: Theoretical Background and Applications Review

**DOI:** 10.3390/s25123577

**Published:** 2025-06-06

**Authors:** Qi-Ang Wang, Ao-Wen Lu, Yi-Qing Ni, Jun-Fang Wang, Zhan-Guo Ma

**Affiliations:** 1 State Key Laboratory for Geomechanics & Deep Underground Engineering, China University of Mining and Technology, Xuzhou 221116, China; zgma@cumt.edu.cn; 2 School of Mechanics and Civil Engineering, China University of Mining and Technology, Xuzhou 221116, China; awlu@cumt.edu.cn; 3 National Rail Transit Electrification and Automation Engineering Technology Research Center (Hong Kong Branch), Hong Kong, China; yiqing.ni@polyu.edu.hk; 4 Department of Civil and Environmental Engineering, The Hong Kong Polytechnic University, Hong Kong, China; 5 MOE Key Laboratory for Resilient Infrastructures of Coastal Cities, College of Civil and Transportation Engineering, Shenzhen University, Shenzhen 518060, China; jf.wang@szu.edu.cn

**Keywords:** Bayesian network, structural health monitoring, damage prediction, data fusion, uncertainty

## Abstract

With accelerated urbanization and aging infrastructure, the safety and durability of civil engineering structures face significant challenges, making structural health monitoring (SHM) a critical approach to ensuring engineering safety. The Bayesian network, as a probabilistic reasoning tool, offers a novel technological pathway for SHM due to its strengths in handling uncertainties and multi-source data fusion. This study systematically reviews the core applications of the Bayesian network in SHM, including damage prediction, data fusion, uncertainty modeling, and decision support. By integrating multi-source sensor data with probabilistic inference, the Bayesian network enhances the accuracy and reliability of monitoring systems, providing a theoretical foundation for damage identification, risk early warning, and optimization of maintenance strategies. The study presents a comprehensive review that systematically unifies the theoretical framework of BN with SHM applications, addressing the gap between probabilistic reasoning and real-world infrastructure management. The research outcomes hold significant theoretical and engineering implications for advancing SHM technology development, reducing operational and maintenance costs, and ensuring the safety of public infrastructure.

## 1. Introduction

With the increasing age of infrastructure and the increasing environmental influences, structural health monitoring (SHM) has become a key research direction in the field of civil engineering [1]. SHM aims to use various sensing technologies and data analysis methods to monitor the status of engineering structures in real time. SHM can timely detect potential damage or performance degradation, ensure the safety of structures, and extend their service life [2]. In this context, the Bayesian network [3], with its unique probabilistic reasoning and decision support capabilities, provides a powerful analytical tool for SHM.

The Bayesian network is a mathematical tool based on the probabilistic graph model, which is famous for its characteristics such as probabilistic modeling, visual representation, comprehensive information, and dynamic update [4]. It describes the system by establishing a probabilistic relationship model between variables and makes use of probabilistic reasoning methods for analysis and prediction. The Bayesian network represents dependencies between variables in the form of directed acyclic graphs, and random variables and conditional probabilities between them through nodes and edges. Nodes in the Bayesian network represent random variables, and edges represent probabilistic dependencies between variables. The topology of the Bayesian network clearly and intuitively shows the causal relationship and probability transfer path of the structure. In SHM, there are often many uncertain factors, such as measurement errors, environmental changes, etc. The Bayesian network can model and deal with these uncertainties through probabilistic reasoning. Second, the Bayesian network can use prior knowledge, observational data, and domain expert knowledge to form comprehensive reasoning and decision results. By collecting and analyzing large amounts of structural monitoring data, the Bayesian network can help engineers and decision-makers better understand and analyze the health of structures and optimize maintenance strategies. In addition, the Bayesian network can dynamically update [5], which can be adjusted according to the new observation data, timely update the parameters and structure of the model, and improve the accuracy and reliability of the model.

The Bayesian network is widely used in SHM. This study introduces the development and basic principles of the Bayesian network and summarizes the definition, basic ideas, and research significance of SHM. The definition of SHM involves real-time or periodic monitoring of engineering structures to evaluate the integrity and performance status of structures. In this context, the Bayesian network provides us with a powerful tool to integrate multi-source data and perform effective analysis to enable monitoring and prediction of structural health status. The application of the Bayesian network in damage prediction, data fusion, uncertainty modeling, and decision support are discussed in detail through the research status at home and abroad. The following presents a visual analysis of the literature across four key aspects.

**Figure 1:**
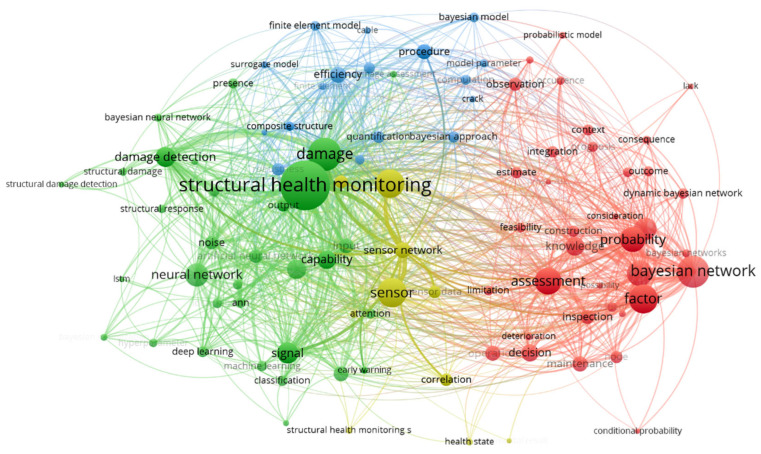
Density distribution map of technological hotspots in structural health monitoring.

**Figure 2:**
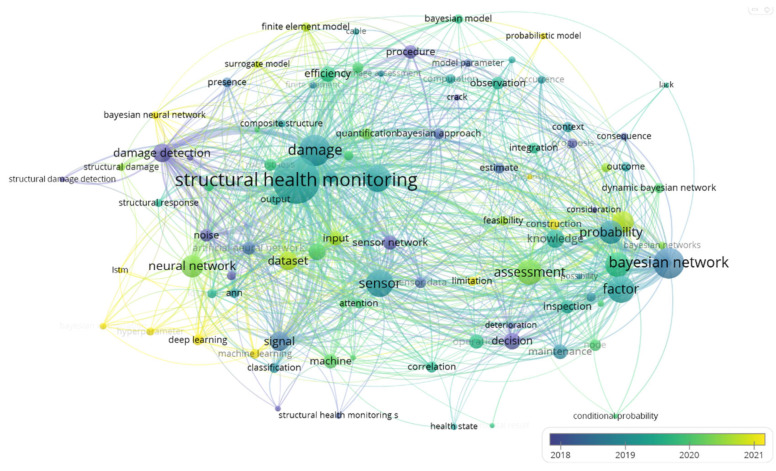
Co-occurrence network visualization of technologies in structural health monitoring.

**Figure 3:**
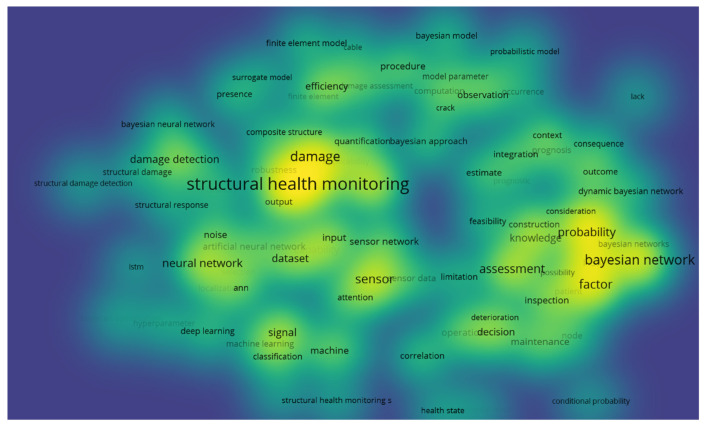
Overlay visualization of technological evolution in structural health monitoring.

The study is based on a co-occurrence analysis of thousands of papers published in the Web of Science database over the past decade, with themes of “Bayesian Network” and “Structural Health Monitoring”, conducted using VOSviewer. The generated visualizations are shown in Figure 1, Figure 2 and Figure 3. Figure 1 shows the relationships between key concepts related to the application of the Bayesian network in SHM. Several main thematic clusters are visible, including SHM, sensors, damage detection, assessment, and the Bayesian network. The Bayesian network plays a crucial role in these areas, particularly in structural assessment and decision support systems, using probabilistic models and data inference to aid in damage prediction and risk evaluation. The dense connections in the diagram highlight how these concepts and methods are closely interconnected in practical applications, contributing to the accuracy and efficiency of SHM.

As shown in Figure 2, the application of the Bayesian network in SHM emphasizes its role in damage prediction, data fusion, uncertainty modeling, and decision support. The connections highlight how the Bayesian network integrates sensor data for more accurate damage detection, models uncertainties in structural conditions, and supports decision-making by providing probabilistic assessments of structural integrity. Over time, the use of the Bayesian network has grown, reflecting its increasing importance in assessing and predicting damage, integrating diverse data sources, and making informed decisions to optimize maintenance and risk management in SHM.

Figure 3 shows a heatmap representing the key concepts related to the Bayesian network in SHM. The heatmap’s intensity and color gradients emphasize the focus areas within SHM, especially in areas like damage prediction, uncertainty modeling, and the use of probabilistic methods for risk assessment and decision support. The bright yellow highlights around certain concepts suggest that they are increasingly important in the research and application of SHM systems.

## 2. An Overview of the Bayesian Network

### 2.1. Overview of Bayesian Network Development

The Bayesian network [6] is a probabilistic graphical model that utilizes Bayes’ theorem to represent conditional dependencies among variables and perform reasoning under uncertainty. As a class of directed acyclic graphs (DAGs), the Bayesian network is widely recognized as one of the most effective frameworks for uncertain knowledge representation and inference in complex systems. The Bayesian network quantifies dependencies between variables through conditional probability tables (CPTs) or distributions (CPDs), where conditional probabilities can be categorized into discrete types (finite-state enumeration), continuous types (e.g., Gaussian distribution modeling), and hybrid types (requiring conditional linear Gaussian models, etc.). The determination method depends on data and knowledge context: expert knowledge can directly define CPTs and validate them through sensitivity analysis; data-driven approaches rely on parameter learning (maximum likelihood estimation, Bayesian estimation) or structure learning (scoring functions, independence tests); hybrid methods integrate both, such as Bayesian updating for dynamically adjusting priors. In practical applications, high-dimensional CPTs require dependency simplification, Monte Carlo sampling quantifies uncertainty, and dynamic systems with time-varying damage necessitate sliding window update mechanisms. These methods collectively support BN in structural health monitoring, ensuring engineering reliability from probabilistic modeling to real-time inference.

Since it was proposed by Pearl in 1988 [7], it has become a research hotspot in recent years. The Bayesian network is a widely used probabilistic-directed acyclic graph model, which uses mature probabilistic calculus theory as the basis for reasoning and processing uncertainty and has been widely used in many fields involving uncertainty [8]. This study mainly discusses reliability engineering. Bayesian network (BN) is a useful framework for engineering reliability assessment. Taking into account the uncertainties associated with component performance and the hazards that components face, the Bayesian network model component states as random variables and captures probabilistic dependencies between component and system performance. Furthermore, in an environment where information is constantly being updated, for example, inspections provide new evaluations of the current state of components, and any information entered into the Bayesian network is propagated through the network to update the evaluation of the system. Finally, the Bayesian network, as a graphical framework, supports clear modeling of the system to facilitate end-user adoption [9]. Using the Bayesian network for reliability assessment in engineering is a hot research area. Many researchers have put their eyes on this field and conducted a lot of research, in which many excellent research results have been born. Over the past decade, several authors have summarized previous studies and written review articles. Wang et al. [10,11,12,13,14,15] investigated Bayesian methods for reliability analysis, data modeling, uncertainty analysis, as well as structural damage detection. Helge [16] and Luigi carried out a systematic review of the reliability assessment of the Bayesian network, focusing on the modeling framework. Kabir [17] and Papadopoulos reviewed the applications of the Bayesian network and Petri networks (PN) in system security, reliability, and risk assessment, highlighting some advantages of Bayesian network-based and PN approaches over other classical approaches. Huang et al. [18] summarized the basic principles of the Bayesian analysis and calculation and then reviewed the latest practice of Bayesian reasoning in the identification and damage assessment of civil infrastructure systems. The focus is on addressing the challenges posed by system identification and damage assessment of civilian infrastructure. In addition, other popular research directions of Bayesian network, such as non-parametric Bayesian network, continuous Bayesian network [19], and fuzzy Bayesian belief networks [20], are proposed by Miguel et al. [21].

### 2.2. Bayesian Network Mathematical Model

According to the classical definition by Pearl [7], a Bayesian network N is represented as an ordered pair N=(G,Θ), where G denotes the structure and Θ represents the network parameters. In graph theory, the DAG G=(X, E) encodes the independent relationships among a set of variables. X corresponds to the non-empty set of all nodes in the graph, defined as $X=\{X_{1},X_{2},\ldots ,X_{r},\ldots ,X_{n}\}$, where each $X_{i}$ can be either an observed variable or a latent variable. The set E comprises directed edges between variables in the DAG and $X_{j}\to X_{i}$ indicates a direct dependency relationship between nodes.

(1) $$E=\left\{X_{j}\to X_{i}\;|\;X_{j}\in \mathrm{pa}(X_{i}),\;i=1,2,\ldots ,n\right\}\;$$

In Equation (1), $\mathrm{pa}(X_{i})$ denotes the “cause” of the node $X_{i}$, also referred as the set of parent nodes.

**Figure 4:**
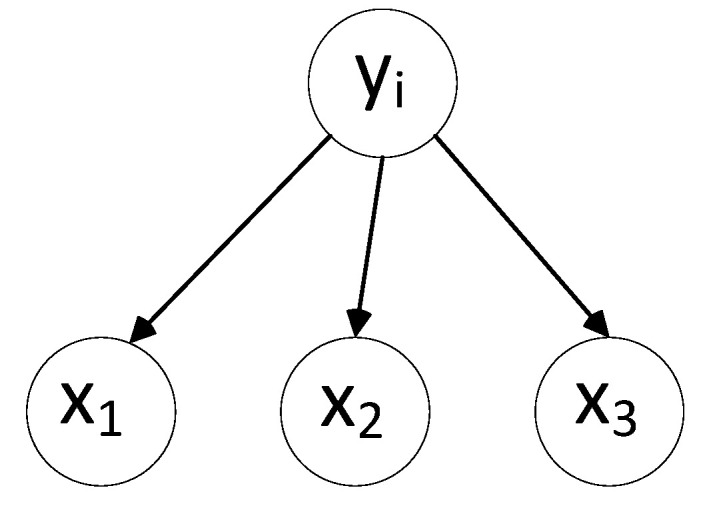
Bayesian network splintering.

The conditional independence assumption of attributes states that the variables x are mutually independent. In the graph representation, as shown in Figure 4, they are reflected by the absence of arcs between the x nodes, such as naive Bayes [22], which can be described in Equation (2).

(2) $$P(x_{1},x_{2},x_{3},y_{j})=p(y_{j})\cdot p(x_{1}|y_{j})\cdot p(x_{2}|y_{j})\cdot p(x_{3}|y_{i})$$

The Naive Bayes classifier utilizes Bayes’ theorem to predict the class of a data point. In classification problems, given a data point (composed of multiple features), the Naive Bayes classifier estimates the probability of the data point belonging to each class and assigns it to the class with the highest posterior probability.

**Figure 5:**
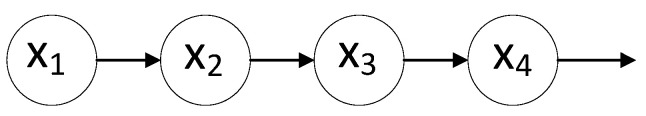
Bayesian network cascades.

The Markov assumption states that a variable x depends solely on the preceding k variables, such as the first-order Markov hypothesis [23], the current variable depends only on the previous variable, which can be described in Equation (3) and Figure 5.

(3) $$p(x_{1},\ldots ,x_{N})=p(x_{1})\prod_{n=2}^{N}p(x_{n}|x_{n-1})$$

For a first-order Markov chain, suppose there is a sequence of states $\left(x_{1},\ldots ,x_{n}\right)$, where each $\left(x_{n}\right)$ represents a state at a point in time (t). The first-order Markov hypothesis states that given the current state $\left(x_{n}\right)$, the conditional probability of the future state $\left(x_{n+1}\right)$ depends only on $\left(x_{n}\right)$, and $\left(x_{1},\ldots ,x_{n+1}\right)$ is irrelevant.

The conditional independence assumption among variable sets, as exemplified in the Bayesian network, posits that the current variable x depends only on its parent nodes. The corresponding mathematical formulation is provided in Equation (4).

(4) $$P(x_{1},\ldots ,x_{n})=\prod_{i=1}^{n}P\left(x_{i}|\mathrm{parents}(x_{i})\right)$$

**Figure 6:**
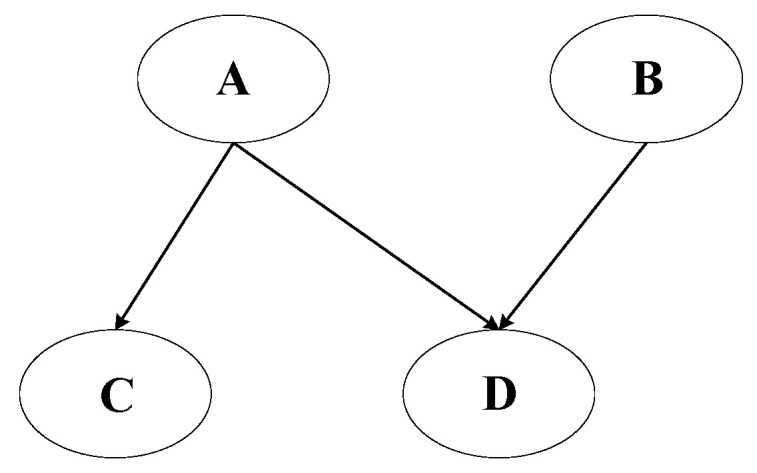
Bayesian network model.

Figure 6 shows an intuitive illustration of the Bayesian network model. The probabilities for each node in the diagram are shown in Table 1, Table 2, Table 3 and Table 4. The Bayesian network depicted in the figure consists of four variables: *A*, *B*, *C*, and *D*. In this network, *A* serves as a parent node, directly influencing both *C* and *D*. Additionally, *B* is conditionally dependent on *A*, as indicated by the directed edge from *A* to *B*. *D* is influenced by both *A* and *B*, reflecting a bidirectional dependency between these variables. *C*, on the other hand, is solely dependent on *A*. This structure suggests that *A* is an independent variable, while *B* and *D* are conditionally dependent on *A*, with *D* also being influenced by *B*. The joint probability distribution of nodes can be expressed as Equation (5). When *D* is given, *A* and *B* are independent of each other.

(5) P(A,B,C,D)=P(C|A)P(D|A,B)P(A)

## 3. Structural Health Monitoring

### 3.1. Definition of Structural Health Monitoring

SHM encompasses diverse definitions in the literature. Gharehbaghi et al. [24] critically reviewed its conceptual foundations, defining SHM as ‘the strategy and process of damage identification and characterization of engineering structures. This definition emphasizes the integration of sensing technologies and analytical methods to assess structural integrity systematically. Structural damage refers to the change in structural material parameters and their geometric characteristics. The process of SHM involves the acquisition of structural response using a sensor array with periodic sampling [25], the extraction of damage-sensitive indicators, and the statistical analysis of damage-sensitive indicators to determine the current structural health status.

SHM technology originated in the 1950s with the initial purpose of load monitoring of structures. With the increasingly large-scale, complex, and intelligent development of structures, the content of structure monitoring technology is gradually enriched, which is no longer simple load monitoring, but the development of structural damage detection, damage assessment, structural life prediction, and even automatic repair of structural damage [26,27,28]. The premise of SHM is to extract parameter signals that can reflect structural characteristics from engineering structures, such as stress, strain, temperature, deformation, speed, acceleration, displacement, and other local or global signals, and then use reasonable and effective information processing methods to extract structural damage and aging information from the collected original data to monitor the load borne by the structure and the operation status of the structure. It provides a reference for the safe use and maintenance of the structure, and achieves the purpose of reducing the maintenance cost, predicting the occurrence of catastrophic events, and reducing the loss to a minimum. This technology has been widely used in aerospace, machinery, and other fields [29,30], but in the field of civil engineering, especially in building structures, it is still in the basic exploration stage.

From the mid–late 1980s to the 1990s, research on SHM systems developed rapidly. Some countries in Europe and the United States first put forward the new concept of SHM, and successively installed health monitoring systems on some important long-span Bridges or Bridges with novel structural systems, mainly monitoring environmental loads, structural vibration, and local stress states [31,32,33]. It is used to monitor construction quality, verify design assumptions, and evaluate structural safety status. With the development of the social economy, people pay more and more attention to disaster prevention and reduction, and the research of SHM systems has become a hot research direction in aerospace, national defense, composite materials, civil engineering, and other fields. All countries are adding health monitoring systems to new and existing important engineering structures [34,35].

This study holds that SHM refers to the strategy of monitoring and identifying structural damage in engineering structures. SHM technology is a comprehensive technology, involving a variety of fields, including civil engineering, dynamics, materials science, sensing technology, computer technology, network communication technology, and other reform research directions.

### 3.2. Approach and Significance of Structural Health Monitoring

SHM involves the use of on-site sensor systems and related analytical techniques to monitor the behavior and performance of structures, including their operability, safety, and durability under all operating conditions. Advanced data analysis methods, such as AI-based intelligent data analysis, are employed to determine structural parameters and damage conditions. When monitoring standards are exceeded, appropriate alarms are triggered for structural performance assessment and damage prognosis [36,37,38]. SHM also supports the prediction of structural health levels and lifespans, providing decision-making support for interventions such as maintenance, refurbishment, and replacement.

Civil engineering structures used for a long time will always deteriorate during their use due to various natural and human factors. With the increase in service time of civil engineering structures, the pressure on structural maintenance work also increases, and so SHM technology comes into being. Its research and application are of great significance to ensure the safe operation of public facilities. Its main research significance is as follows: (1) Improve safety performance: SHM can effectively detect the damage and abnormal status of buildings and other structures, allowing for timely maintenance and reinforcement measures to improve their safety performance and reduce the occurrence of unexpected accidents [39]. (2) Reduce maintenance costs: The SHM technology can realize real-time monitoring and remote diagnosis and can help reduce the maintenance costs [40]. (3) Driving technological progress: SHM involves multiple disciplines, including structural engineering, sensor technology, data acquisition, and signal processing. It has great significance in promoting the development and innovation of related technologies [41]. (4) Enhance public trust: SHM can achieve real-time monitoring of buildings and other structures, improve public trust in public facilities, and enhance the reliability of public facilities [42].

To summarize, the research and application of SHM technology has important social and economic significance and will have a positive impact on ensuring the safe operation of public facilities, promoting technological progress, and improving public welfare [43,44,45].

## 4. Application of Bayesian Network in Structural Health Monitoring

The Bayesian network, as a probabilistic graphical model, has been widely used in SHM for its unique capability to extract causal relationships from multi-sensor data and quantify uncertainties in fault diagnosis and prediction [46,47,48]. As shown in Table 5, unlike deep learning methods (e.g., LSTM and CNN) that excel in automated feature extraction for complex temporal or spatial patterns (e.g., crack propagation trends or vibration spectrograms), BN prioritizes interpretability and explicit uncertainty modeling, making it preferable for risk-sensitive decision support (e.g., maintenance prioritization under budget constraints). However, BN faces challenges in handling high-dimensional dynamic systems compared to LSTM-gated architecture, which efficiently captures long-term dependencies in sensor time series. Additionally, traditional machine learning methods (e.g., SVM and random forests) offer computational efficiency for small-scale classification tasks (e.g., binary damage detection) but lack BN’s ability to fuse domain knowledge with probabilistic reasoning. Future advancements may focus on hybrid frameworks, such as embedding BN-based uncertainty quantification into deep neural networks, to balance interpretability, scalability, and predictive accuracy in SHM applications. The following sections detail key applications of the Bayesian network in SHM.

### 4.1. Application of Bayesian Network in Damage Identification

The Bayesian network can be applied to structural damage identification [49,50,51]. The Bayesian network can establish an association model between damage and monitoring data, judge whether there is damage to the structure by inferring the real-time monitoring data, and further determine the location and degree of the damage. This helps to quickly identify structural problems, take timely maintenance and enhancement measures, and ensure the stability and reliability of the structure [52,53,54].

With the continuous development and the application of sensors, Nguyen [55] proposed the application of a Bayesian inference network based on a distributed sensor network and multi-sensor data flow in the damage detection of composite plate holes. Based on the data of the distributed sensor network, the Bayesian network was established to detect hole damage in composite materials. The performance of the Bayesian network is then checked by diagnostic validation of use case corruption. Lampis [56] et al. studied how to apply BBN to diagnose system faults. First, a fault tree (FT) is constructed to indicate how component failures combine to cause unexpected deviations in the variables monitored by the sensor. Converting the Fourier transform to BN creates a model that represents a single network made up of subnetworks, and the posterior probability of component failure gives a measure of the components that cause the observed symptoms.

Lampis [56] proposed a Bayesian network procedure that can be generalized to any system in which a causal relationship structure can be established between the state of the system components and the readings of the sensor. On this basis, Conde [57] proposed a reverse analysis program to investigate the causes of pathological conditions of masonry arch Bridges using Bayesian methods. The study formulated damage investigation as a parameter estimation problem and developed a nonlinear finite element model to simulate load scenarios and the initial undamaged configuration of the bridge. Computer model predictions were compared with actual measurement data to obtain a distribution of the most likely parameter values for reproducing existing damage patterns. The posterior probability distribution of unknown parameters was estimated using simulation techniques, in particular the Markov chain Monte Carlo (MCMC) method. The computational burden is reduced by using a Gaussian process simulator. The method was tested in a real case study of a masonry arch bridge in the village of Kakodiki, Greece, and the results showed that the method can accurately reproduce existing damage patterns.

**Figure 7:**
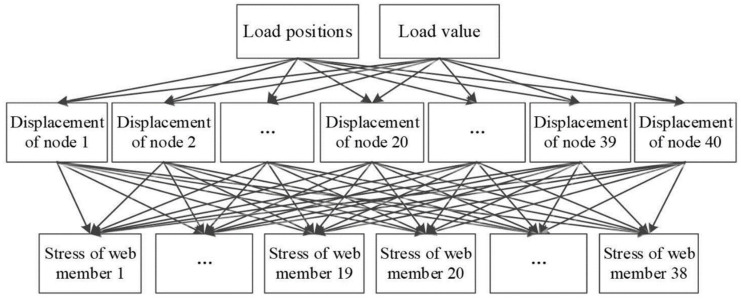
The GBN structure [60].

Subsequently, various Bayesian network-based approaches for damage prediction have emerged in the research field. As an illustration, Laura’s study [58] introduced a Transfer Bayesian Learning (TBL) technique specifically designed for SHM of historical buildings. This approach focused on probabilistic classification of processed data through selective extraction of key information components. The structural agent model (SM) served as the foundation for establishing multi-category classifications according to particular mechanical parameters sensitive to damage. Practical implementation occurred at Italy’s Consoli Palace, where sensor-equipped monitoring systems continuously collected SM data that interacted with physical observations. A sensitivity damage chart (SDC) functioned as the classification criterion. Through Bayesian updating of damage parameters, probabilistic damage assessment became achievable, with validation conducted via numerical simulations of potential damage scenarios involving vibration patterns, thermal variations, and crack development. Finite element nonlinear analysis helped pinpoint regions vulnerable to damage. In another significant contribution, Alazzawi’s research [59] developed an innovative technique employing raw structural response time-series data combined with deep residual networks (DRN) for precise condition assessment. The integration of residual learning algorithms with Bayesian optimization enhanced network performance, enabling accurate determination of both damage extent and position. This breakthrough marked the pioneering application of DRN to SHM non-image datasets, particularly for electromechanical impedance (EMI) signal analysis, representing a substantial advancement in monitoring methodologies. Regarding Gaussian Bayesian networks, Sun’s work [60] demonstrated their implementation using strain monitoring data for damage evaluation in steel truss bridges. As shown in Figure 7, the developed methodology constructed a three-tier GBN framework incorporating load parameters, structural deformation, and stress measurements. The model architecture consisted of load factors as primary nodes, structural deflections as secondary nodes, and truss element stress data forming the tertiary layer. Training procedures involved finite element simulations under varying load conditions, followed by parameter optimization through maximum likelihood estimation techniques.

### 4.2. Application of Bayesian Network in Data Fusion

At the 9th International Conference on Information Fusion in 2006 [61], researchers formulated the target tracking based on received signal strength in the sensor networks using Bayesian network representation. Data fusion among the same type of sensors in an active sensor neighborhood is referred to as cross-sensor fusion, conceptualized as “cooperative fusion”. Subsequently, at the Third International Conference on Advanced Design and Manufacturing Engineering in 2013, Zhang [62] proposed a selective incremental information fusion method based on the Bayesian network, which enables the fusion algorithm to actively select the most relevant information and decisions and enables the fusion model to adapt to the dynamic changes in the external environment. The sensor information selection, fusion, and decision were integrated into the Bayesian network. In the same year, at the 16th International Conference on Information Fusion, Park [63] presented a hybrid (both discrete and continuous variables) Multi-Entity Bayesian Networks (MEBN) learning algorithm, a MEBN that combines first-order logic with Bayesian network for representing and reasoning uncertainty in complex, knowledge-rich domains.

In recent years, Bayesian networks have fused data from different sensors to improve the accuracy and reliability of SHM systems. By integrating different types of sensor data into a unified probabilistic framework, networks can more fully assess the health of structures and provide more reliable monitoring results. Ramin [64] proposed a novel mathematical architecture for conditional and operational risk monitoring of complex engineered systems (CES). Given the complexity of the operational data and the complexity of the system itself, the challenge of risk and reliability analysis at CES was to perform and update risk and reliability assessments on the entire system with a high frequency. The proposed architecture integrates Bayesian network (BN) and deep learning (DL) models to address data and system complexity in a single architecture that provides system-level insights. BN was used to model systems, subsystem relationships, and scenarios leading to adverse events, and to fuse subsystem-level information. DL models were trained for subsystem diagnosis based on state monitoring data, and their outputs were integrated into the root node of BN. Matteo [65] proposed a method to update conditional probability tables in Bayesian belief networks by fusing expert knowledge and system monitoring data, aiming to continuously update CPT by incorporating new data-driven evidence about system behavior. The method was tested on the health status assessment of Bridges and demonstrated the improved accuracy and diagnostic capability of BBN analysis.

To improve the accuracy and efficiency of the Bayesian network in practical applications, researchers continue to improve and develop. GAO [66] introduced the idea of parallel ensemble learning and proposed a new hybrid Bayesian network structure learning algorithm. The algorithm adopted the elite-based structure learner using a genetic algorithm (ESL-GA) as the base learner. L. Zou [67] proposed a convolutional network based on Bayesian optimization and the channel fusion mechanism. The network used convolutional autoencoders to extract compressed features and reconstruct input data. The channel fusion mechanism was introduced to reduce the error of reconstructed input data. Li [68] proposed a novel distributed Bayesian data fusion algorithm, for arbitrary periodically connected communication graphs. To bridge the research gap of Bayesian network data fusion in SHM, Ierimonti [69] proposed an original framework comprising the following key stages: (i) select the possible damage scenarios to be monitored by SHM; (ii) reproduce the selected damage scenarios in a numerical model of the bridge; (iii) perform the Bayesian Model Class Selection among the selected scenarios; (iv) update the information on the Bayesian network (evidence and/or conditional probabilities), previously assembled with all the possible input variables (SHM, visual inspections and so on) for evaluating the risk of bridge failure.

### 4.3. Application of Bayesian Network in Uncertainty Modeling

The Bayesian network can effectively deal with uncertainty, which is crucial in SHM. Structure monitoring data is often affected by noise, error, and uncertainty, and the Bayesian network can model and deal with these uncertainties through probabilistic reasoning to provide more reliable monitoring results and predictions. Different models have different performances in dealing with different aspects of uncertainty. Ding [70] proposed a system-level fatigue reliability assessment model based on the Bayesian network, which regarded the bridge deck as a parallel system. The study highlighted the importance of probabilistic models such as the Bayesian network in assessing fatigue reliability due to uncertainties in monitoring data and model interpretation. It provided a numerical case study with three scenarios involving different stress amplitudes and cycle number distributions in the weld, demonstrating the impact on fatigue life reliability. The Monte Carlo method was used to solve complex integrals in the Bayesian network, which provided a practical method for evaluating fatigue performance.

The method proposed by Li [71] utilized a deep neural network model, which consists of a convolutional layer for feature extraction and a long short-term memory (LSTM) layer for time series prediction. The LSTM cells were modified to introduce randomness into some of the cell parameters, and Bayesian inference was used to estimate the probability distribution of the network parameters. The method was applied to a railway bridge under a high-speed train load to verify its effectiveness. Zhang [72] proposed a Bayesian neural network (BNN) method for probabilistic model updating using incomplete modal data in structural engineering applications. The research focused on improving the reliability of finite element models through proxy modeling, especially the efficient updating of structural parameters using BNN. The BNN framework allowed the quantification of uncertainty in the estimated parameters by utilizing the nonlinear relationship between the selected parameters and the incomplete modal data. The method included an adaptive sampling strategy based on truncated Gaussian distribution to optimize the update parameters. Numerical examples and applications on laboratory and experimental structures demonstrated the accuracy and efficiency of the proposed framework for quantifying parameter uncertainty in structural model updating. Chen [73] introduced a two-stage stochastic model updating method for highway bridges based on long rail strain sensing. It addressed the need for accurate assessment of bridges due to the rapid growth of bridges and the limitations of existing model update methods. By combining radial basis function neural networks with Bayesian theory, the method aimed to improve the efficiency and accuracy of bridging model updates. The feasibility of this method was proved by numerical examples and laboratory model experiments. The Stochastic model update (SMU) method considered the uncertainty and provided the probability density distribution of modified parameters, which provided a more accurate and robust basis for structural reliability assessment. Xiao [74] discussed the establishment of a pavement deterioration probability prediction model based on a Bayesian neural network (BNN). By combining Bayesian theory and neural networks, a probabilistic model based on BNN was developed to predict pavement deterioration using data from Shaanxi Province, China. The BNN-based model not only maintains high prediction accuracy comparable to deterministic neural network models but also incorporates uncertainty, making it more reliable in predicting pavement deterioration and helping engineers make maintenance decisions.

**Figure 8:**
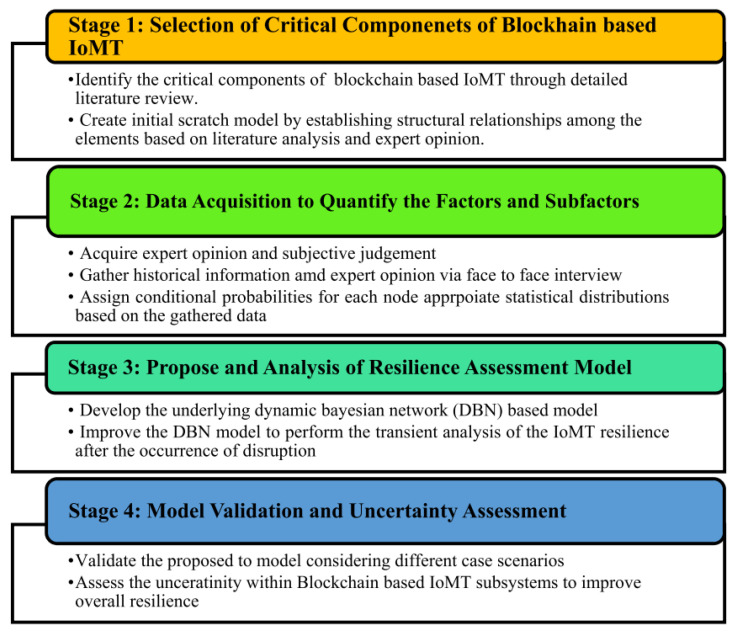
Proposed approach for resilience assessment using DBN [76].

To provide more reliable monitoring results and predictions, various new methods are being constantly proposed and applied. Cheng [75] proposed a deep Bayesian survival method to estimate the service life of railway tracks. The study highlighted the importance of reliable estimation of track life for the predictive maintenance of railway systems. The proposed method utilized a deep neural network to capture the nonlinear relationship between covariates and rail life. Monte Carlo leaks were incorporated into deep neural networks to provide confidence intervals for estimated lifetimes, allowing uncertainty to be quantified. The method was implemented and evaluated on a four-year dataset of a section of railway track in Australia. The results showed that the proposed method is superior to other commonly used models, achieving a consistency index of 0.80 (*C* index) and providing an accurate estimate of the service life of the railway track. As shown in Figure 8, Shah [76] proposed a dynamic Bayesian network (DBN) model to evaluate the elasticity of blockchain-based Internet of Medical Things (IoMT) systems at different time intervals, considering the evolution characteristics of the relevant variables. The model captured time dependence and used an information theory approach to mitigate uncertainties in elastic properties. Zhang [77] proposed a new method to apply Bayesian models on average to multiple prediction models to account for the uncertainties of models and parameters. The results showed that the Gonzalez–Sagaseta model is the best for predicting surface subsidence, while the Loganathan–Poulos model is the best for predicting vertical and horizontal underground deformation. Wang [78] introduced a quantitative risk assessment model of heavy goods vehicle (HGV) tunnel fires based on functional resonance analysis (FRAM) and a Bayesian network (BN). The study aimed to identify the mechanisms and key risk factors of tunnel fires involving HGV and quantify the risk using probabilistic analysis. The integration of FRAM and BN provided a comprehensive approach for analyzing and evaluating the formation and evolution of tunnel fire incidents while addressing uncertainties in risk assessment.

**Figure 9:**
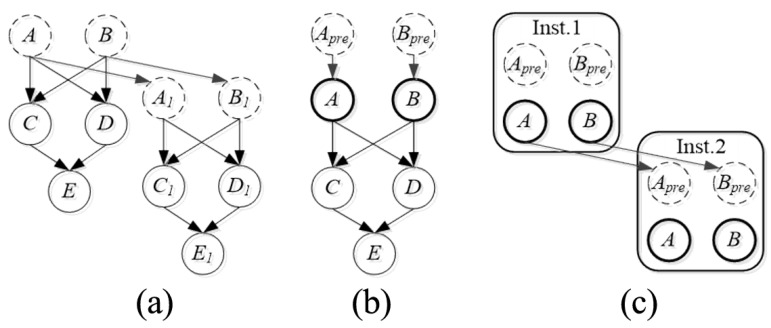
Concept of an OOBN used in a DBN: (**a**) DBN, (**b**) Class, and (**c**) OOBN [79].

In addition, when the structure being evaluated is too complex to be modeled with the Bayesian network, especially when the structure consists of the same or similar components, it is complicated to build the Bayesian network-based reliability evaluation model. As shown in Figure 9, an object-oriented Bayesian network (OOBN) was produced. OBBN was a suitable tool for evaluating the reliability of objects with large, complex, and hierarchical structures [79]. For example, Wang [80] proposed a structural reliability prediction method for steel bridge members based on a dynamic target-oriented Bayesian network (DOOBN), which can effectively simulate the process of steel bridge members and predict their structural reliability over time. Liu et al. [81] proposed a new method to model the risk management process for complex systems.

### 4.4. Application of Bayesian Network in Decision Support

The Bayesian network can also be used to aid decision-making [82,83]. Based on the accurate assessment and prediction of structural health status, the network can guide structural maintenance and repair and can help decision-makers formulate the best maintenance strategy and resource allocation scheme [84,85,86].

Rita [87] proposed a method to systematically assess and manage risks associated with tunnel construction. The method involved combining a geological prediction model, which allows the prediction of geology before tunnel construction, with a construction strategy decision model, which allows one to select among different construction strategies the one that leads to the least risk, both of which are based on the Bayesian network. This risk assessment method provided a powerful tool for planners and engineers to systematically assess and mitigate the inherent risks associated with tunnel construction. In terms of bridge deterioration monitoring, in 2018, Matteo [88] et al. proposed a new Bayesian belief network (BBN) method for bridge deterioration monitoring. First, he proposed a method for constructing BBN structures and defining conditional probability tables. Then, using evidence of bridge behavior (such as bridge displacement or acceleration due to traffic) as input for the BBN model, the probability of the health state of the entire bridge and its elements is updated, and the degree of deterioration is monitored. Finally, the finite element model of a steel truss bridge is taken as an example to demonstrate the effectiveness of the proposed method.

Since then, the probabilistic framework of the Bayesian network has been rapidly developed and applied to realistic risk analysis, and different Bayesian network frameworks have been proposed. Enrico [89] et al., proposed a probabilistic framework based on the Bayesian network for updating the risk of bridge aftershocks to reduce the uncertainty in assessing the risk of bridge failure. Park et al. [90] proposed a damage grade classification criterion to establish the correspondence between the calculated damage value D and the actual observed damage. BN was used to describe the probabilistic relationship between various random variables involved in earthquake damage assessment, such as earthquake-induced ground motion intensity, bridge response parameters, earthquake damage, etc. The framework was applied to a hypothetical bridge. The results showed that the accelerometer and visual information have a significant impact on bridge damage estimation and thus affect the decision under the threat of future aftershocks. This field currently lacks a statistical knowledge and parameter system that is easy to quantify.This study provided practitioners with a realistic approach to risk assessment and further understanding of dynamic and stage-related risks in the life cycle of large infrastructures. The framework can be modified and used for other real-world risk analyses where risks are complex and develop in stages [91]. On this basis, Gibson [92] also proposed the Bayesian network (BN) framework to simulate the seismic failure model of earth–rock dam systems in the Central and Eastern United States (CEUS). The proposed BN framework utilized graphical representations of dependencies to facilitate risk-based decision-making. The framework included a Hazard BN for modeling seismic hazards and a dam system BN for evaluating dam performance and failure modes. Miguel [93] also introduced a method to evaluate the structural criticality of bridges in a network due to extreme traffic loads. The proposed approach utilized the Bayesian network and binary connection functions for long-term site-specific simulations using recorded traffic data. The structural response generated by simulated traffic was evaluated and the extreme value of traffic load effects was obtained. By comparing the extreme load effect with the design load effect, the critical state of the bridge was obtained, and the result was visualized. This method was applied to the national highway network and compared with the simplified method. This method provided a valuable tool for assessing the status of bridge networks and making wise bridge management decisions.

## 5. Conclusions

**Figure 10:**
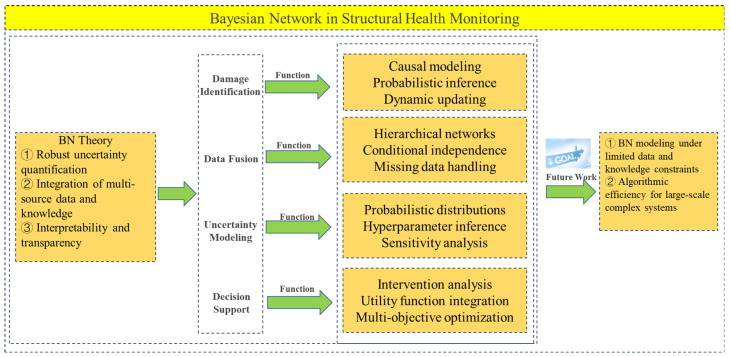
Summary of the main research contents of this study.

As a probabilistic graph model, Bayesian networks have a wide application prospect in the field of SHM. As shown in Figure 10, the study presents an overview of the development and fundamental principles of the Bayesian network, summarizes the definition, core concepts, and research significance of SHM, and provides a detailed review of the recent applications of the Bayesian network in engineering. These applications include damage prediction, data fusion, uncertainty modeling, and decision support. In terms of damage prediction, by monitoring the state data of the structure at different time points, the Bayesian network can analyze the evolution process of damage and predict possible damage in the future. In the application of data fusion, the Bayesian network can fuse data from different sensors to improve the accuracy and reliability of SHM systems. In the application of uncertainty modeling, structure monitoring data is often affected by noise, error, and uncertainty, and the Bayesian network can model and process these uncertainties using probabilistic reasoning, to provide more reliable monitoring results and predictions. In terms of decision support, based on accurate assessment and prediction of structural health, the network can guide structural maintenance and repair, and help decision-makers to formulate the best maintenance strategy and resource allocation plan.

Although Bayesian networks have demonstrated significant potential in structural health monitoring (SHM) through applications like damage prediction, data fusion, and decision support, their practical implementation remains constrained by core challenges such as strong data dependency, high computational complexity, and the trade-off between interpretability and accuracy. Current research predominantly relies on high-quality prior knowledge and sufficient training data to build reliable inference models. However, in real-world engineering scenarios, sparse sensor data, incomplete historical records, and environmental disturbances collectively lead to probabilistic model biases, particularly in time-varying system modeling like Dynamic Bayesian Networks (DBNs), where increasing node counts trigger the curse of dimensionality, severely compromising real-time performance. Future research should focus on multidisciplinary collaborative innovation. On one hand, adaptive model updates could be achieved through reinforcement learning integration, or federated learning frameworks could be employed to reconcile the conflict between data scarcity and privacy protection. On the other hand, it is essential to develop edge computing-optimized BN algorithms (such as FPGA-based hardware acceleration) to enable real-time monitoring of resource-constrained devices. Simultaneously, expanding BN uncertainty quantification capabilities will support risk-driven maintenance planning by converting probabilistic outputs into actionable engineering decision-making criteria. These breakthroughs will not only enhance BN robustness in SHM applications but also facilitate its integration into smart infrastructure ecosystems, providing both theoretical and technical support for full lifecycle management.

## Figures and Tables

**Table 1:** Probability table of node *A*.

*A*	*P*(*A*)
True	0.1
Not true	0.9

**Table 2:** Probability table of node *B*.

*B*	*P*(*B*)
True	0.4
Not true	0.6

**Table 3:** Probability table of node *C*.

*A*	*P*(*C*)	
	True	Not True
True	0.8	0.1
Not true	0.2	0.9

**Table 4:** Probability table of node *D*.

*A*	*P*(*D*)			
	*B* = True		*B* = Not True	
	True	Not True	True	Not True
True	0.8	0.6	0.6	0.3
Not true	0.2	0.4	0.4	0.7

**Table 5:** Comparison between the BN method and other methods.

Method Type	Core Strengths	Main Drawback
BN	Highly interpretable, supporting causal reasoning Explicitly quantifies uncertainty Can integrate expert knowledge with data	High computational complexity (curse of dimensionality) Reliance on prior knowledge and structured data Dynamic modeling requires extensions (DBN)
LSTM	Strong sequential modeling capability, capturing long-term dependencies Automatic feature extraction, adapting to complex temporal patterns	Black-box models, poor interpretability Requires large amounts of labeled data High computational resource consumption
Traditional machine learning	High computational efficiency, suitable for small samples Some models are interpretable (e.g., decision trees) Robust to noise	Reliant on manual feature engineering Difficult to handle high-dimensional temporal or spatial data Unable to quantify uncertainty
Deep learning	Automatically extract unstructured features (such as images, spectrograms) High precision, suitable for complex pattern recognition	Data demand is extremely high, and labeling costs are substantial Lacks the ability to model uncertainty High complexity in model deployment
